# Chemical characterization of wheat-based waste derived from a pharmaceutical process for its potential valorization

**DOI:** 10.3389/fchem.2024.1437221

**Published:** 2024-11-13

**Authors:** Lidia Ciriaco, Luana Izzo, Giulia Graziani, Maria Grazia Ferraro, Marialuisa Piccolo, Roberto Ciampaglia, Barbara Maglione, Roberta Palladino, Simone Albarella, Eugenia Romano, Alberto Ritieni, Carlo Irace, Paolo Grieco

**Affiliations:** ^1^ Department of Pharmacy, School of Medicine and Surgery, University of Naples Federico II, Naples, Italy; ^2^ Department of Molecular Medicine and Medical Biotechnology, University of Naples Federico II, Naples, Italy; ^3^ BioChem Lab, Department of Pharmacy, School of Medicine and Surgery, University of Naples Federico II, Naples, Italy; ^4^ Farmaceutici Damor, Naples, Italy; ^5^ Department of Pharmacy, University of Naples Federico II, Naples, Italy

**Keywords:** Triticum vulgare, pharmaceutical waste, biocompatibility, bioactive compounds, upcycling, UHPLC Q-orbitrap HRMS

## Abstract

**Introduction:**

We report the analysis and characterization and the preliminary biological evaluation, of both liquid and solid wastes obtained from the processing of wheat (*Triticum vulgare*) to produce the most iconic phytostimulin-based pharmaceutical products. The study aims to verify whether the waste can be reused in another process and not destined to its simple destructive disposal.

**Methods:**

In this perspective, we first carried out an in-depth chemical-physical analysis of the waste together with a biocompatibility evaluation to plan the feasible final choice of waste destination. The liquid and solid waste derived from the processing of wheat extract were analyzed and characterized through ultra-high-performance liquid chromatography coupled with high-resolution Orbitrap mass spectrometry (UHPLC-Q-Orbitrap HRMS).

**Results:**

Results highlight that ferulic acid represent the most abundant phenolic compound for solid waste with a content of 89.782 mg/kg and dihydroferulic acid is the predominant for liquid waste (6.24 mg/L). These concentrations represent 55.87% and 84.39% of the total concentration of bioactive compounds for liquid and solid waste, respectively. The antioxidant activity registered for the solid extract was 8.598 and 7.262 mmol trolox/kg, respectively for ABTS and FRAP assays. The total phenolic content (TPC) in the liquid extract undergoes a significant percentage reduction compared to the solid waste. As regards toxicity, both liquid and solid wastes were investigated *in vitro* preclinical models of human skin (HaCaT cells and HDFa) after 24, 48, and 72 h of exposure. No cytotoxic effect was noted even at the highest tested concentration (100 μg/mL) at 72 h.

**Discussion:**

Overall, considering its chemo-physical features and active ingredients, we believe that this waste is highly reusable as a starting material for the development of cosmeceutical products. Thus, this study allows us to motivate the destination of the waste of the production in a recyclable raw material for additional industrial processes, thereby promoting an eco-friendly circular economy operation.

## 1 Introduction

Waste derived from pharmaceutical processes is a significant problem worldwide, due to the negative effects it has on the environment, the economy, and society. In the literature, there are several scientific studies whose aim is to analyze and evaluate waste management (Sapkota and Pariatamby, 2023). Pharmaceutical waste is generated and accumulated along the various stages and practices of the production chain. The accumulation of enormous quantities of waste and by-products, not only from food and agriculture but also pharmaceuticals, often containing precious bioactive compounds, constitutes a problem of considerable interest due to its environmental impact (Vilas-Boas et al., 2021). Reducing the formation of by-products in work processes or valorizing them for the purposes of other production processes, represents today the main option to avoid environmental problems and help the economy and society. Today, various biotechnological and nanotechnological approaches are available that can be used for the valorization of by-products of natural origin with the aim of improving their potential applications (Freitas et al., 2021). Biologically active natural molecules and phytochemical compounds can be obtained directly through an extraction process rather than using a synthetic process starting from chemicals (Lemes et al., 2022). Although the extraction process allows to obtain the precious biocomponents used for therapeutic and cosmetic purposes, the destination of the wastes generated has to be considered. Wastes indeed consist of both the residues of the natural raw material and the solvents used in the extraction process.

In this context, the standard practice of “reduce, reuse and recycle” is a good strategy aiming to reduce waste in landfills, preserving natural resources and thus reducing environmental pollution (Madaan et al., 2024). Although the practice “reduce, reuse, and recycle” is useful for reducing general waste and each method has its advantages, the reuse should be the first imperative when waste comes from industrial processes. Upcycling is an innovative and creative way to give new life to old or unwanted products and materials by adding value to the product, with important benefits for the environment, society, and the economy (Sneh et al., 2024).

Upcycling helps create a circular economy, where materials can constantly be reused instead of turning into waste. This is important because it allows single-use items to be in use indefinitely. For a circular economy to be effective, manufacturers need to consider this feedback loop in the design and production of products to ensure they can be upcycled in the future.

In this scenario, the goal of our work is to valorize the use of the waste generated from the aqueous extract of the Triticum Vulgare (TVE). TVE has been proven to have biological regenerative properties useful in the wound healing. Furthermore, its processing is a simple water-based extraction procedure with not potentially toxic or harmful solvents. Thus, it can be easily ascribed to a green approach for the recovery of bioactive compounds with a reduced environmental impact (Sanguigno et al., 2018; Tito et al., 2020; Romano et al., 2023). The production of the phytostimulin-based product (Fitostimoline^®^, Farmaceutici Damor S.p.A., Naples, Italy) starting from wheat follows a management philosophy based on the responsible concept of reduction-reuse-recycling, aimed primarily at the sustainable management of all active processes. Although the production is not particularly excessive to create disposal problems, we were interested in analyzing this waste to verify whether it could find a more noble alternative use given that the process that leads to its production is green. Valorization and recycle of waste require an in-depth analysis in physical and chemical terms, together with the subsequent evaluation of its potential usefulness as raw material for other processes, economic feasibility, and adherence to environmental and legislative sustainability. Moving in this direction, the evaluation of biocompatibility and safety profile in human cellular models adds further value to the possibility of waste reuse. The ever-growing amount of waste from pharmaceutical activities is leading to the necessity of identifying through physicochemical analysis and biological evaluations new ways to recycle waste as raw materials in further applications. Nowadays, waste products from industrial processes have a significant economic impact due to their high presence within the European Union (Idiano et al., 2024). The aim of this work is to (i) perform a chemical characterization of bioactive compounds in both liquid and solid wastes obtained from the processing of wheat (*Triticum vulgare*) and (ii) evaluate the safety profile in human cellular models to propose this resource as a source of active components.

## 2 Materials and methods

### 2.1 Chemicals and reagents

Methanol (MeOH), ethanol (EtOH), water (UHPLC-MS grade), acetonitrile (UHPLC-MS grade), chloroform, hydrochloric acid, glacial acetic acid, formic acid (FA were purchased from Merk (Milan, Italy), whereas deionized water (<18 MX cm resistivity) was provided from a Milli-Q water purification system (Millipore, Bedford, MA, United States). The standards of polyphenols (purity >98%) were acquired from Merck group (Milan, Italy). The compound of 2,2-azinobis (3-ethylbenzothiazoline-6-sulphonic acid) diammonium salt (ABTS), ferrous chloride (FeCl_2_), 1,1-Diphenyl-2-picrylhydrazyl (DPPH), Folin–Ciocalteu reagent, Sodium carbonate anhydrous, potassium persulfate, 2,4,6-Tris (2-pyridyl)-*s*-triazine (TPTZ), (±)-6-Hydroxy-2,5,7,8-tetramethylchromane-2-carboxylic acid commonly called Trolox, gallic acid were acquired from Sigma Aldrich (Milan, Italy).

### 2.2 Sampling

*Triticum vulgare* was obtained through a standard manufacturing protocol of germination, under restricted conditions of temperature, light and humidity. The aqueous extract of Triticum vulgare (TVE) is produced using a newly developed extraction method patented by Farmaceutici Damor (Riccio, 2018), using a simple water-based extraction procedure with not potentially toxic or harmful solvents. Thus, it can be easily ascribed as a green approach for the recovery of bioactive compounds with a reduced environmental impact. In more detail, liquid extraction was performed from the germ wheat macerated and forced under 300 atm of pressure. The liquid sample was recovered through an ultrafiltration membrane filter with a cut-off below 30 kDa.

Wastes derived from the production process were then collected appropriately. Solid waste (SW) was generated from the exhausted solid-phase pressed while liquid waste (LW) was recovered after the ultrafiltration process. These wastes were provided by the company Farmaceutici Damor. SW was dried (to allow homogenization), ground, and subjected to analysis to investigate the profile of the polyphenolic component. Both solid and liquid wastes were analyzed for their phenolic components in different ways. LW was analyzed for evaluation of the qualitative-quantitative profile of soluble polyphenolic compounds, after filtration on 0.2-micron nylon filters and diluted with 1:10 (*v/v*) in methanol.

### 2.3 Polyphenolic extraction on solid waste

The polyphenolic compounds on solid waste were extracted following a procedure reported in the literature for the sprouted wheat, with little and opportune modifications (Van Hung et al., 2011). Free phenolics were extracted using 80% chilled ethanol as described in literature (Van Hung et al., 2009). Shortly, 10 mL of 80% chilled ethanol were added to a 15 mL centrifuge tube containing 1.0 g of dried sample. The content was vortex mixed and tumbled for 20 min at a room temperature before centrifugation at 4000 *g* for 5 min. The supernatant (free phenolic extracts) was collected. The residue was re-extracted twice with 10 mL of 80% chilled ethanol and all supernatants were combined. The supernatant was dried using a nitrogen evaporator (Laborata 4000; Heidolph Instruments Italia Srl, Milan, Italy) and then reconstituted with 10 mL of methanol. The extracts were then stored at −18 °C under nitrogen until analyzed.

### 2.4 Determination of total phenolic content (TPC)

The Folin-Ciocalteu method was used for determining the total phenolic content in accordance with the procedure reported by Izzo et al. (2020). Briefly, 500 µL of deionized water and 125 µL of the Folin-Ciocalteu reagent 2 N were added to 125 µL of extract. The tube was mixed and incubated for 6 min in dark conditions. Then, 1.25 mL of 7.5% of sodium carbonate solution and 1 mL of deionized water were added. The reaction mixture was maintained in dark conditions for 90 min. Finally, the absorbance at 760 nm was measured through a spectrophotometer system. Results were expressed as mg of gallic acid equivalents (GAE)/g of dry weight sample for solid waste and as mg of gallic acid equivalents (GAE)/mL of extract for liquid waste.

### 2.5 UHPLC-Q-orbitrap HRMS analysis

The qualitative and quantitative profile of bioactive compounds were performed by UHPLC (Dionex UltiMate 3000, Thermo Fisher Scientific, Waltham, MA, United States) equipped with a degassing system and an autosampler device. Analysis chromatographic was carried out with a thermostat (T = 30°C) using the Accucore aQ column (particle size 2.6 µm, 100 × 2.1 mm), (Manufacturer: Thermo Fisher Scientific). The mobile phase consisted of 0.1% acetic acid glacial in water (A) and Acetonitrile (B). The injection volume was 5 µL. Metabolites of interest were eluted by setting a flow rate of 0.4 mL/min. The gradient elution program was as follows: 0–5 min-5% B phase, 25 min-40% B phase, 25.1 min- 100% B phase, 27 min- 100% B phase, 27.1 min- 5% B phase, 35 min- 5% B phase. The total run time was 35 min, and the flow rate was 0.4 mL/min.

Mass spectrometry was performed on a Q-Exactive Orbitrap system equipped with an electrospray ionization (ESI) source operating in negative mode. The flow rates of the sheath gas, auxiliary gas and sweep gas were set at 35 arbitrary units (arb unit), 10 arb unit, respectively, with the spray voltage set to −2.8 kV under negative mode. The capillary temperature was set at 275°C, and the auxiliary gas heater temperature was 350 °C. The S-lens RF level was set at 50 V. Detection was achieved considering the exact mass with a mass error <5 ppm. Data analysis was performed using Xcalibur software 3.1.66.19. (Xcalibur, Thermo Fisher Scientific, Waltham, MA, United States).

### 2.6 Antioxidant activity

The antioxidant activity of the liquid waste and the extract of polyphenols obtained by the solid waste was assessed spectrophotometrically by using two different assays, namely, FRAP and ABTS. The obtained data were expressed as millimoles of Trolox Equivalents (TE)/kg of dry weight sample for solid waste and as millimoles of Trolox Equivalents (TE)/L for liquid waste.

#### 2.6.1 ABTS assay

Free radical-scavenging activity was measured by using the method reported by Luz et al. (2018). Briefly, 9.6 mg of 2,2-azinobis (3-ethylbenzothiazoline-6-sulphonic acid) diammonium salt was solubilized in 2.5 mL of deionized water (7 mM) and 44 µL of solution of potassium persulfate (K_2_S_2_O_8_; 2.45 mM) were added. The solution was kept in dark conditions at room temperature for 16 h prior to use. Afterward, ABTS^⋅+^ solution was diluted with ethanol to reach an absorbance value of 0.70 (±0.02) at 734 nm. Then, to 1 mL of ABTS^⋅+^ solution with an absorbance of 0.700 ± 0.050, 0.1 mL of opportunely diluted sample was added. After 2.5 min wait, the absorbance was immediately measured at 734 nm. Results were expressed as mg of gallic acid equivalents (GAE)/g of dry weight sample for solid waste and as mg of gallic acid equivalents (GAE)/mL of extract for liquid waste.

#### 2.6.2 FRAP assay

The antioxidant capacity of samples was estimated spectrophotometrically following the procedure of Benzie and Strain (1996). The ferric reducing/antioxidant power (FRAP) reagent was prepared by mixing acetate buffer (0.3 M; pH 3.6), TPTZ solution (10 mM), and ferric chloride solution (20 mM) in the proportion of 10:1:1 (v/v/v). Freshly prepared working FRAP reagent was used to perform the assay. Briefly, 0.150 mL of the appropriately diluted sample was added to 2,850 mL of FRAP reagent. The value of absorbance was recorded after 4 min at 593 nm. The method is based on the reduction of Fe^3+^ TPTZ complex (colorless complex) to Fe^2+^-tripyridyltriazine (intense blue color complex) formed by the action of electron-donating antioxidants at low pH. Results were expressed as millimoles of Trolox Equivalents (TE)/kg of dry weight sample for solid waste and as millimoles of Trolox Equivalents (TE)/L for liquid waste.

### 2.7 Proximate composition analysis of solid waste

#### 2.7.1 Water (moisture)

Water content was established via air oven drying (103°C) of samples of solid waste (5 g each per sample) until constant weight. Water values were calculated through the difference between initial weight of the sample and the constant weight of the sample, after drying process. The percent moisture was calculated by the following formula:

$$\%\text{ moisture}=\left(w_{1}-w_{2}\right)\;{100/w}_{1}$$

Where w_1_ = initial weight of sample; w_2_ = final weight of sample (after drying).

The results are expressed as percentage of water (Stadlmayr et al., 2020).

#### 2.7.2 Fat content evaluation

Fat extraction was carried out following the method of Folch et al. (1957) with some modifications. In brief, 1.0 g of sample was combined with 20.0 mL of a mixture chloroform/methanol (2:1 *v/v*) in a screw-top glass tube. The tubes were then shaken for 30 s before being shaken for 15 min in an orbital shaker at room temperature (∼25°C). Next, the tubes were centrifuged at 1500 *g* for 15 min to then thoroughly decant the bulk of the chloroform/methanol solution, ensuring that all solid material remained at the bottom of the tube. The upper layer was covered, and the residue was extracted another time with 20.0 mL of mixture and the process was repeated. Finally, all the mixture were collected (about 40 mL) and washed with 10 mL of water. The chloroform phase was dried, and the residue was weighed. The percent crude fat was determined by using the following formula:

% crude fat=Wt of chloroformic extract × 100/Wt of sample

The fat content was then expressed as g/100 g sample.

#### 2.7.3 Protein content evaluation

The quantitative determination of protein was carried out using the Kjeldahl, 1883, later modified by Gunning and Arnold (Pickel, 1915). In more detail, the procedure involved an initial step of mineralization of the total nitrogen in the sample, leading to the formation of ammonia, which was subsequently distilled and titrated. Concerning the volume of hydrochloric acid used, the nitrogen content is calculated. This value was then multiplied by a constant (5.7 for cereals), which allowed us to obtain the protein content value expressed as the percentage of protein per 100 g of dry sample.

#### 2.7.4 Ash evaluation

The sample was calcined at 550°C for 2–4 h. The appearances of gray-white ash indicate complete oxidation of all organic matter in the sample. The weight loss by calcination gave the total organic matter content of the sample. The residue is the mineral part or ash (Stadlmayr et al., 2020).

### 2.8 Minerals content evaluation

Microwave digestion was performed for the determination of other elements in the solid sample. 500 mg of sample was transferred to a TFM®PTFE vessel with 6 mL of 65% ultrapure concentrated HNO_3_ (14.33 mol/L) and 1 mL of 30% H_2_O_2_. The sample was subjected to digestion using a microwave (MW-AD, Ethos EZ Microwave Digester, Milestone, Shelton, CT, United States). The heating program for digestion consisted of 4 phases: phase 1 (90°C for 7 min), phase 2 (170°C for 5 min), phase 3 (210°C for 5 min) and phase 4 (210°C for 20 min). In all phases, the power was set to 1000 W. The sample was resuspended with 25 mL of bi-distilled water to undergo analysis by graphite furnace atomic absorption spectrometry (GFAAS). Analyses for arsenic, boron, calcium, chromium, cobalt, iron, manganese, selenium, copper, and zinc were performed by atomic absorption spectroscopy (AAS) according to the AOAC International, 1995. An AA-6300 atomic absorption spectrophotometer (Shimadzu, Columbia, MD, United States), equipped with an ASC-6100 autosampler (Shimadzu, Columbia, MD, United States) and a GFA-EX7i graphite furnace atomizer (Shimadzu, Columbia, MD, United States) was used. Instrument control and data analysis were performed using Multi-Element Program software (WizAArd software, Shimadzu, Columbia, MD, United States). Argon was used as the carrier gas. The AAS instrument is equipped with a hollow cathode lamp for linear sources and a deuterium lamp as a background corrector. Graphite pyrolytically coated tubes were used for the atomization step. To optimize the analytical signal, various tests were performed with different lamp intensities, sample injection volumes, and temperature ranges (1600◦C–1800°C for atomization).

Prior to analysis, a calibration line was performed for each analyte to be searched for using the multi-element standard prepared at concentrations of 0 μg/L (Cal Blk), 4 μg/L (Cal Std 1), 12 μg/L (Cal Std 2) and 20 μg/L (Cal Std 3). For the determination of other elements in the liquid sample from the *T. vulgare* extraction process, the microwave extraction step was not carried out, but dilutions and subsequent analysis by graphite furnace atomic absorption spectrometry (GFAAS) was performed. The results are reported in Table 3.

### 2.9 Cell cultures

To evaluate the biological effects and biocompatibility profile of samples under investigation, specific bioscreens were performed on preclinical skin models by using primary and continuous cultures of both epidermal and dermal origin, representing ideal models to study cellular responses to *in vitro* treatments as well as toxicological responses.

HaCaT cells, an immortalized, nontumorigenic human keratinocyte cell line, were received from CNR (courtesy of Dr Valeria Cicatiello) to accomplish *in vitro* bioscreens. HaCaT were cultured in Dulbecco’s modified Eagle’s medium (DMEM; Invitrogen, Paisley, United Kingdom) containing high glucose (4.5 g/L), supplemented with 10% fetal bovine serum (FBS; Cambrex, Verviers, Belgium), L-glutamine (2 mM; Sigma, Milan, Italy), penicillin (100 U/mL; Sigma), and streptomycin (100 lg/mL; Sigma) at 37°C in a humidified 5% CO_2_ atmosphere, according to ATCC recommendations (Miniaci et al., 2016). Human adult dermal fibroblasts (HDFa) were acquired by ATCC after isolation from the skin of a white male donor (PCS-201–012™). They were cultured in Fibroblast Basal Medium (ATCC) supplemented with recombinant human fibroblast growth factor (rh FGF, 5 ng/mL), L-glutamine (7.5 mM), ascorbic acid (50 μg/mL), hydrocortisone hemisuccinate (1 μg/mL), rh Insulin (5 μg/mL) and fetal bovine serum (FBS, 2%). Penicillin-Streptomycin-Amphotericin B Solution (Penicillin: 10 Units/mL, Streptomycin: 10 μg/mL, Amphotericin B: 25 ng/mL) was also added. HDFa cells were seeded at a density between 2,five to five × 10^3^ cells/cm^2^ and have been passed when approximately 80%–100% confluence was reached in actively proliferating cultures. Cells were cultured in a humidified 5% carbon dioxide atmosphere at 37°C, according to supplier’s recommendations (Gazzillo et al., 2023).

### 2.10 Preparation of biological samples (LW and SW) for *in vitro* experiments

The different waste samples were subjected to distinct treatment methods based on their respective characteristics. Liquid waste (LW) samples were freeze-dried, while solid waste (SW) samples were extracted using the procedure for sprouted wheat (Van Hung et al., 2011), with some modifications. The resultant extract obtained from solid waste was dried using a rotavapor, reconstituted with 10 mL of water (UHPLC-MS grade), and subsequently freeze-dried.

### 2.11 Bioscreens *in vitro*

Cellular responses to samples application *in vitro* were investigated through the estimation of a “cell survival index”, arising from the combination of cell viability evaluation with automated cell count (Irace et al., 2017). The cell survival index is calculated as the arithmetic mean between the percentage values derived from the MTT assay and the automated cell count. HaCaT and HDFa were inoculated in 96-well culture plates at a density of 10^4^ cells/well and allowed to grow for 24 h. The medium was then replaced with fresh medium, and cells were continuously treated for 24, 48 and 72 h with a range of concentrations (5→100 μg/mL) of biological samples, i.e., liquid waste (LW) and solid waste (SW). Using the same experimental procedure, cells were also incubated with cisplatin (cDDP) as positive control for cytotoxic effects (data not shown). After *in vitro* incubations, cell viability was evaluated using the MTT assay procedure, which measures the level of mitochondrial dehydrogenase activity using the yellow 3-(4,5-dimethyl-2-thiazolyl)-2,5-diphenyl-2H-tetrazolium bromide (MTT, Sigma) as substrate. The assay is based on the redox ability of living mitochondria to convert dissolved MTT into insoluble purple formazan. Briefly, after the treatments, the medium was removed, and the cells were incubated with 20 μL/well of a MTT solution (5 mg/mL) for 1 h in a humidified 5% CO_2_ incubator at 37 °C. The incubation was stopped by removing the MTT solution and by adding 100 μL/well of DMSO to solubilize the obtained formazan. Finally, the absorbance was monitored at 550 nm using a microplate reader (iMark microplate reader, Bio-Rad, Milan, Italy).

Cell number was determined by TC20 automated cell counter (Bio-Rad, Milan, Italy), providing an accurate and reproducible total count of cells and a live/dead ratio in one step by a specific dye (trypan blue) exclusion assay. Bio-Rad’s TC20 automated cell counter uses disposable slides, TC20 trypan blue dye (0.4% trypan blue dye w/v in 0.81% sodium chloride and 0.06% potassium phosphate dibasic solution) and a CCD camera to count cells based on the analyses of captured images. Once the loaded slide is inserted into the slide port, the TC20 automatically focuses on the cells, detects the presence of trypan blue dye and provides the count. When cells are damaged or dead, trypan blue can enter the cell allowing living cells to be counted. Operationally, after treatments in 96-well culture plates, the medium was removed, and the cells were collected. Ten microliters of cell suspension, mixed with 0.4% trypan blue solution at 1:1 ratio, were loaded into the chambers of disposable slides. The results are expressed in terms of total cell count (number of cells per ml). If trypan blue is detected, the instrument also accounts for the dilution and shows live cell count and percent viability. Total counts and live/dead ratio from random samples for each cell line were subjected to comparisons with manual hemocytometers in control experiments. The calculation of the concentration required to inhibit the net increase in the cell number and viability by 50% (IC_50_) is based on plots of data (n = 4 for each experiment) and repeated three times (total n = 12). IC_50_ values were calculated from a dose response curve by nonlinear regression using a curve fitting program, GraphPad Prism 8.0, and are expressed as mean values ±SEM (n = 24) of four independent experiments (Irace et al., 2017; Ferraro et al., 2023).

### 2.12 Cytomorphological analysis

HaCaT cells and HDF were grown on 60 mm culture dishes by plating 5 × 10^5^ cells. After reaching the sub-confluence, the medium was replaced with fresh medium, and cells were continuously treated for 24, 48, and 72 h with a range of concentrations (5→100 μg/mL) of biological samples (LW and SW). After treatments, cells were examined by a phase-contrast microscope (Labovert microscope, Leizt) to monitor any morphological change. Microphotographs at a ×200 total magnification (×20 objective and ×10 eyepiece) were taken with a standard VCR camera (Nikon) (Ferraro et al., 2023).

### 2.13 Statistical analysis

Statistical analysis of data was performed by two-way ANOVA analysis (SPSS 13.0) followed by the Tukey-Kramer multiple comparison test to evaluate significant differences; *p*-values ≤0.05 were considered as significant. All the determinations were undertaken in triplicate, and results were expressed as mean ± standard deviation (SD).

## 3 Results

### 3.1 Quantification of flavonoids and phenolic compounds

The identified polyphenols (n = 16) in both solid and liquid waste were characterized using a UHPLC coupled to a high-resolution Orbitrap mass spectrometry. For the quantitative analysis calibration curves were built in appropriate solvents and regression coefficients higher than 0.990 were obtained. Quantitative analysis showed that the dominant phenolic acid identified was ferulic acid, followed by dihydroferulic acid, apigenin-6-arab-8-hexoside, feruloyl quinic acid, apigenin-C-hexoside-hexoside, chlorogenic acid, caffeic acid and others (Table 1). In the solid waste polyphenols extract, ferulic acid represents up to 55.5% of the total polyphenol content identified. In the liquid waste, dihydroferulic acid showed a reduction by 75% compared to the solid waste. A clear reduction in the content of active compounds of up to 95.4% was observed between the solid and liquid waste.

**TABLE 1:** Polyphenol content in solid and liquid waste. The results are displayed as average value (μg/g) for solid waste and as average (μg/mL) for liquid waste and standard deviation (SD).

Bioactive Compounds	Solid Waste	Liquid Waste
	μg/g dw	μg/mL
Ferulic acid	89.782 ± 1.626	*nf*
Dihydroferulic acid	24.934 ± 1.323	6.24 ± 0.212
Apigenin-C-hexoside-pentoside	12.398 ± 0.372	0.076 ± 0.052
Apigenin-6-arab-8-hexoside	12.167 ± 1.203	0.08 ± 0.055
Feruloyl quinic acid	7.917 ± 0.388	0.039 ± 0.02
Caffeic acid	2.715 ± 0.067	0.716 ± 0.010
Chlorogenic acid	2.299 ± 0.134	*nf*
Kaempferol-3-O-rutinoside	1.722 ± 0.095	0.022 ± 0.008
Coumaric acid-O-hexoside	1.264 ± 0.044	0.03 ± 0.002
Apigenin-C-hexoside-hexoside	1.259 ± 0.081	0.016 ± 0.008
Isoorientin-2-O-rhamnoside	1.259 ± 0.050	0.016 ± 0.007
Kaempferol-3-O-glucoside	0.768 ± 0.001	0.04 ± 0.000
Caffeic acid-O-hexoside	0.663 ± 0.082	*nf*
Naringenin	0.563 ± 0.020	0.037 ± 0.002
Sinapoyl-hexoside	0.528 ± 0.008	0.08 ± 0.002
Apigenin-7-O-glucoside	0.351 ± 0.017	0.002 ± 0.000
Total	160.697	7.394

^a^ dw: dry weight.

### 3.2 Antioxidant capacity and total phenolic content (TPC) of liquid and solid waste

The antioxidant activity of both liquid and solid wastes was assessed using two different assays (DPPH and ABTS assay). The total phenolic content (TPC) was evaluated through the Folin-Ciocalteu test. A calibration curve of inhibition, built with Trolox^®^, was employed as a positive control of the antioxidant assays, while gallic acid was employed to perform the calibration curve for TPC evaluation. Table 2 displays the data as millimoles of Trolox equivalent per kilogram or liter depending on the typologies of the sample is considered solid or liquid (average value and SD). In both antioxidant activity tests a higher antioxidant activity was observed for the solid extract. The total phenolic content in the liquid extract undergoes a significant percentage reduction compared to the solid waste.

**TABLE 2:** Evaluation of the antioxidant activity through ABTS and DPPH assay and the total phenolic content (TPC) (Folin-Ciocalteu Test). The analyses were performed in triplicate and data expressed as mean ± standard deviation.

	ABTS		FRAP		TPC	
	mmol	mmol	mmol	mmol	mg GAE/g	mg GAE/mL
Samples	trolox/kg	trolox/L	trolox/kg	trolox/L		
Solid Waste	8.598 ± 0.053		7.262 ± 0.240		1.771 ± 0.005	
Liquid Waste		0.223 ± 0.011		0.285 ± 0.009		0.105 ± 0.000

### 3.3 Nutritional composition of solid waste

Nutritional components of the solid waste were evaluated using different techniques. The Folch method was used to evaluate the content of fat within the sample. Protein was ascertained through Kjeldahl method. The moisture of the sample was determined to express the data as content of dry material. The solid waste is composed of proteins 3.6 ± 0.3 g per 100 g, fat 2.3 ± 0.6 g per 100 g, and ash 4.1 ± 0.7 g per 100 g. The moisture in the sample was 39% ± 2.4%. The results shown were the average of three repeated measurements and were reported as average ±standard deviation.

### 3.4 Minerals content evaluation

Table 3 reports the results obtained from analysis by graphite furnace atomic absorption spectrometry (GFAAS). The solid waste sample was previously treated by microwave digestion, while the liquid waste was directly diluted and subjected to GFAAS analysis. All analyses were performed in triplicate, and results are expressed as averages of mg/kg and mg/L for solid and liquid waste, respectively, and are reported with standard deviations.

**TABLE 3:** Quantitative determination of minerals in the solid and liquid waste samples.

Minerals	Solid Waste mg/Kg	SD	Liquid Waste mg/L	SD
Ammonia (NH_3_)	9.25	±0.29	6.005	±0.3
Phosphate (PO_4_^3-^)	30.8	±1.1	40.85	±1.05
Calcium (Ca^++^)	29.33	±2.1	18.18	±1
Magnesium (Mg^++^)	18.89	±1.8	14.5	±1.05
Sulphate (SO_4_^2-^)	20.5	±0.8	41.9	±1.25
Arsenic (As^+++^)	0.0078	±0.02	0.01	±0.025
Boron (B)	<0.2	—	<0.2	—
Manganese (Mn^++^)	0.162	—	<0.0001	—
Zinc (Zn^++^)	0.159	±0.016	0.2555	±0.014
Copper (Cu^++^)	0.026	±0.005	0.0095	±0.003
Cobalt (Co^++^)	<0.0001	—	<0.0001	—
Iron (Fe^+++^)	0.0945	±0.004	0.015	±0.002
Selenium (Se)	<0.0001	—	<0.0001	—
Chromium (Cr^+++^)	0.0411	±0.008	0.0762	±0.005
Chloride (Cl^−^)	3.3	±0.13	0.9	±0.065

All the determinations were performed in triplicate and results are expressed as mg/kg and mg/L means for solid and liquid wastes, respectively, and standard deviation.

### 3.5 Bioactivity evaluation in preclinical models of human skin

**FIGURE 1:**
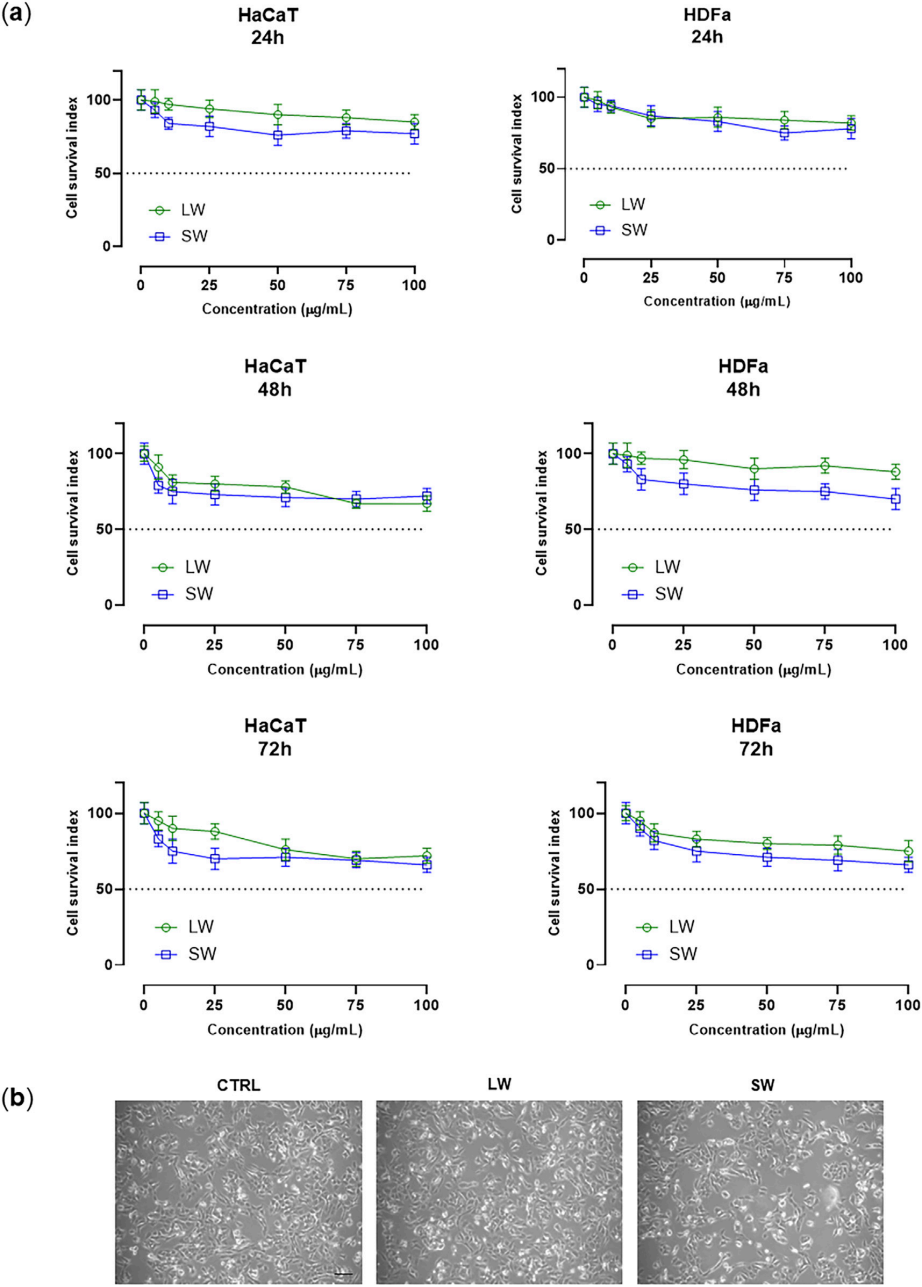
**(A)** Cell survival index, evaluated by the MTT assay and live/dead automated cell count, for HaCaT cells and HDFa following 24, 48, and 72 h of incubation with the indicated concentrations (0–100 μg/mL) of liquid waste (LW) and solid waste (SW), as reported in the legend. Data are expressed as a percentage of untreated control cells and are reported as mean of four independent experiments ±SEM (n = 24). The Cell Survival Index was calculated as described in the experimental section and plotted in line-graphs against the different concentrations of the tested samples. **(B)** Representative photomicrographs of HaCaT cells monolayers at ×200 total magnification (×20 objective and ×10 eyepiece) by phase-contrast microscopy after 48 h of treatment with 100 μg/mL of LW and SW, as indicated in the legend.

Both liquid (LW) and solid (SW) wastes were investigated for their potential toxicity by well-established *in vitro* preclinical models of human skin. Epidermal and dermal cultures were selected for biocompatibility analysis of the biological samples under investigation Piccolo et al. (2019). Quantitative (i.e., automated cell counts) and functional (i.e., mitochondrial redox activity) assays allowed for the assessment of cellular responses to *in vitro* treatments (Ferraro et al., 2021). Overall, by targeted bioscreens a “Cell survival index” to characterize cellular viability and responses to experimental conditions was established, as fully described in the experimental section (Irace et al., 2017). Representative concentration-effect curves in the examined cellular models (HaCaT cells and HDFa) after 24, 48, and 72 h of exposure to the indicated concentrations (µg/mL) of the biological samples (LW and SW) are reported in Figure 1A. As can be clearly observed, bioscreens allow the examined samples to be defined as fully biocompatible. Indeed, no significant interference with cell viability, growth, and proliferation was observed. In line, no cytotoxic effect was noted even at the highest tested concentration (100 μg/mL) of both LW and SW following prolonged incubation times (72 h). Consequently, IC_50_ values calculated from concentration-effect curves are all higher than 100 μg/mL, indicative of a good safety profile in human cells. In addition, cytomorphological analyses on do not show noteworthy alterations in cellular morphology (Figure 1B).

## 4 Discussion

Recently, have been reported several studies aimed at valorizing the waste obtained from the natural matrix in the context of the upcycling economy and reduce the environmental pollution. The analysis and characterization of the phenolic compounds contained in aqueous extracts of fennel waste, pea pods and from spent coffee grounds have been already performed (Castaldo et al., 2021a; Castaldo et al. 2021b; Castaldo et al. 2021c).Valid examples of optimization of biofuel production with reduction of final waste have been already reported in literature, demonstrating that a green production process brings not only environmental but also economic benefits for companies (Sarkar et al., 2024; Mridha et al., 2024). In this context, we were motivated to analyze waste generated from the production of the aqueous extract of the Triticum Vulgare (TVE) with the aim of promoting an eco-friendly circular economy operation.

The specific extract of *T. vulgare* (TVE) is manufactured by a recently implemented extraction process (Riccio, 2018) of Farmaceutici Damor, and it is used as the active principle in pharmaceutical formulations for the treatment of decubitus ulcers, skin lesions, and burns. The company Farmaceutici Damor produces about 2–3 tons of waste each year, which compared to the production data is a relatively small amount. However, the cost of disposing of this waste could be onerous. As reported above, the extraction process adopted for the preparation of one of the most iconic pharmaceutical products based on phytostimulins, is green as it is based on the use of ecological solvents characterized by a high extraction capacity. The aqueous extract of *T. vulgare* (TVE) was obtained through a simple water-based extraction process with non-potentially toxic or harmful solvents. The use of a green extraction process in production represents an essential point for the valorization of a by-product or waste and for its potential use in a subsequent production process. The solid and liquid waste analyzed in this study represents approximately 7.5% and 5% of the entire production process. Therefore, for the entire production process, a total loss between solid and liquid of 12.5% is estimated in terms of yield of the finished product. The ratio between the solid fraction and the liquid fraction in terms of mass is of the order of 1.5 to 1.

The approach of exploiting a by-product or waste obtained as a result of an industrial process to obtain a phytochemical preparation would not only significantly reduce waste, but could also create new sources of income, thus supporting the circular economy. Ensuring environmental and economic sustainability is crucial, especially if a technical-economic assessment of the benefits obtained is carried out. From an economic and environmental point of view, to enhance the by-products of the processing of natural products, the characterization of the chemical composition and the analysis of the chemical-physical characteristics are fundamental (Espro et al., 2021).

Phenolic compounds are widely distributed in plants and have recently gained much attention due to their antioxidant activity and ability to scavenge free radicals with potential beneficial implications for human health (Muscolo et al., 2024). Phenolic compounds can be classified as secondary plant metabolites, formed by plants during their secondary metabolism (Susanti et al., 2024). Many of them are bioactive and can have antioxidant, antimicrobial, anticarcinogenic and many other beneficial health effects.

Wheat is an important component of the human diet and, therefore, may be an important source of phenolic antioxidants. In particular, ferulic acid is the predominant phenolic acid found in wheat bran, constituting approximately 90% of the total phenolic compounds (Li et al., 2024).

This observation pushed us to evaluate the by-products of wheat processing from a chemical-physical point of view to understand the potential of its reuse from a circular economy perspective. Literature shows that phenolic acids in wheat grains are mainly present in a form bound to other cereal components such as starch, cellulose, β-glucan, and pentosan (Yu et al., 2001; Vaher et al., 2010). Insoluble bound phenols can be released by basic, acidic, or enzymatic treatment of samples prior to extraction (Liyana-Pathirana and Shahidi, 2006). The identification of the main chemical constituents and the quantification of the phenolic component, almost always present in nature and endowed with antioxidant activity, represents the starting point that can favor its potential industrial exploitation.

The good antioxidant activity of the analyzed wastes, mainly due to the presence of phenols, demonstrates the possibility of recovering or reusing the phytochemical components contained in these by-products for other production processes.

The antioxidant activity of the extract obtained by solid waste is associated, as already well known for wheat, mainly with ferulic acid (Zhou et al., 2004; Klepacka and Fornal, 2006). The quali-quantitative analysis via UHPLC Q-Orbitrap HRMS of the two fractions of processing waste, i.e., solid and liquid, highlighted a different composition of the phenolic fraction. In fact, while in solid waste the phenolic fraction is mainly represented by ferulic acid, in the liquid component of waste the main phenolic component is made up of dihydroferulic acid.

Although solid and liquid waste are quite comparable in quantitative terms, the analysis carried out indicates that their chemical content is very different. This significantly affects their actual reuse value for other production processes. In fact, the in-depth chemical-physical analysis and characterization reported in this study highlighted that, regarding the nature of the phenolic substances, the solid component is mainly rich in ferulic acid (on average 90 μg/g in dry weight) and dihydroferulic acid (on average 24 μg/g in dry weight) while the liquid component is mainly represented by dihydroferulic and caffeic acid, among other things in quite limited quantities (on average 6.24 μg/mL and 0.7 μg/mL, respectively).

Comparing the results of our study with recent literature concerning the reuse and characterization of industrial waste derived from various matrices, several notable differences arise, primarily attributable to the differing biological matrices and extraction methodologies employed. Nevertheless, the existing literature presents numerous examples of waste reuse that is rich in bioactive compounds.

The investigation conducted by Sibhatu et al. (2021) explored the utilization of brewery spent grain (BSG) residues for the extraction of ferulic acid, a naturally occurring antioxidant. This extraction was optimized through an acid treatment followed by alkaline hydrolysis using sodium hydroxide (NaOH), achieving a maximum yield of 46.17 mg/100 g of ferulic acid from BSG, as determined by the Box-Behnken design.

Concentrations of ferulic acid and other active ingredients are lower than those reported in the cited studies; in fact, the data indicate that the solid component is mainly rich in ferulic acid (90 mg/kg dry weight) and dihydroferulic acid (24 mg/kg dry weight), while the liquid component contains mainly dihydroferulic acid and caffeic acid, although in relatively limited concentrations (6.24 mg/L and 0.7 mg/L, respectively). However, it is worth highlighting that the polyphenol extraction method used in our study (Van Hung et al., 2011) does not use acids or bases and is a more environmentally friendly process.

In relation to the study on fennel waste conducted by Castaldo et al. (2021a), the extract demonstrated a ferulic acid concentration of 258 mg/kg but constituting only 4.43% of the total. In contrast, analysis of coffee waste (Castaldo et al., 2021c) revealed a significantly lower ferulic acid content than our waste, 0.8 mg/kg compared to 89.78 mg/kg identified in our solid waste, indicating a ferulic acid concentration that is 100 times higher in our samples. Despite this disparity, coffee waste showed a potential application in the preparation of fortified biscuits.

It is imperative to underscore that, in terms of bioactive compounds, particularly within polyphenolic extracts, it is essential to consider the full spectrum of compounds present, as they may exert synergistic effects. Consequently, we contend that the extraction of polyphenols without the isolation of a single bioactive compound is more advantageous, especially in light of our objective to adhere to principles of the circular economy, which advocate for the minimization of the number of steps and processes involved. The analysis carried out gives solid waste a higher recovery value than liquid waste due to the higher phenol content. In fact, ferulic acid contained mainly in solid waste could find use as a raw material in other production processes. For example, in the natural production of vanillin with biotechnological means and not with synthetic means, thus obtaining a product that can be labeled as “natural” and which could be an alternative both to the product obtained by extraction and to the additive obtained by synthesis, with costs of decidedly more contained production (Xu et al., 2024).

In this context, evaluations conducted in preclinical models of human skin allow both solid and liquid waste from the processing of wheat (*Triticum Vulgare*) to be defined as highly biocompatible. Indeed, cellular responses observed after *in vitro* treatments indicate an excellent safety profile of the wastes under investigation, at least in skin-derived cell. Of note are outcomes obtained in human primary cultures (HDFa) deriving directly from human skin, which represent preclinical cellular models very sensitive to xenobiotic effects and thereby particularly suitable for toxicology studies. Overall preclinical tests confirm the possibility of reusing and valorizing wastes from the industrial process to produce the phytostimulin-based therapeutics, paving the way for their potential upcoming applications in the biomedical and cosmetic field. Considering the positive feedback, we have attained in human skin cells further endorses a possible use of these materials in cosmetics, as well as raw material for the preparation of formulations for topical applications.

Recently, the use of waste was proposed for the development of cosmetic formulations. In particular, extracts obtained from clementine peels and olive leaves show to endowed 25% of antioxidant activity and no cytotoxic effects (*in vitro* studies on NCTC2544 Cells). Creams with these ingredients are stable at various temperatures and safe for human use, demonstrating good spreadability properties and pseudoplastic behaviour, promoting a circular economy and reducing environmental impact (d’Avanzo et al., 2024). In a study by Weronika et al. (2024), they investigated the potential of defatted strawberry seeds, obtained after oil extraction, as a source of phenolic compounds with cosmetic applications. The extracts, rich in tiliroside, kaempferol 3-glucoside and ellagic acid, were obtained with a mixture of water and ethanol. Chemical analyses showed antioxidant properties and cytoprotective activity against oxidative stress in human fibroblasts. The defatted strawberry seeds could therefore be used as beneficial additives for skin care products. Additionally, in a study reported by Draghici-Popa et al. (2024), grape marc, a by-product of the wine industry, they explored the use of grape-seed polyphenolic extracts to produce sunscreens. Extracts, obtained from different grape varieties (Merlot, Fetească Neagră, Blaufränkisch, Isabella) using ethanol and acetone solutions, were analyzed for polyphenol content, antioxidant activity and sun protection factor (SPF). The best extract, from Merlot grape seeds with 70% ethanol, showed an SPF of 7.83, with stable and safe creams.

Based on this evidence reported in literature, the cosmeceutical applications represent one of a possible and potential use of the active ingredients contained in this waste. Moving in this direction, the herein-described outcome can be propaedeutic to further in-depth cellular studies aimed at a precise characterization of biocompatible waste recycling in the biomedical field. Certainly, one of the most economical valorization strategies for the reuse of this processing waste is to evaluate its potential use in the production of food supplements, and antioxidants, or used as a phytoprotective and anti-aging ingredient for cosmetic products. Therefore, the analysis and characterization of waste resulting from the production of a pharmaceutical preparation starting from wheat seeds can be seen as a strategic approach to a circular bioeconomy, where economic and practical advantages deriving from the valorization of waste can guarantee efficient recycling with a positive environmental impact.

## 5 Conclusion

This study allowed us to shed light on the potential characteristics of these wastes and to establish the basis for future investigations on their alternative applications, with particular attention to the solid waste containing the highest phenol component represented by ferulic acid. This result confers to the analyzed solid waste a potentially useful value as for other wastes containing phenolic compounds previously identified and reported in literature.

Although the waste obtained from the industrial process to produce phytostimulin-based pharmaceutical products is not particularly excessive to create disposal problems, the analysis aimed to verify whether it could find a more noble use. In fact, for the characteristics identified with the analysis carried out, this waste could represent a starting resource in other areas such as cosmeceuticals or food.

However, further studies are needed to validate its real applications as a base component of cosmeceutical products, since ferulic acid is a commonly recognized antioxidant and used to protect the skin from damage caused by free radicals.

## Data Availability

The raw data supporting the conclusions of this article will be made available by the authors, without undue reservation.

## In Memoriam

Dedicated to the memory of Alberto Ritieni.
